# Library of molecular associations: curating the complex molecular basis of liver diseases

**DOI:** 10.1186/1471-2164-11-189

**Published:** 2010-03-20

**Authors:** Stefan Buchkremer, Jasmin Hendel, Markus Krupp, Arndt Weinmann, Kai Schlamp, Thorsten Maass, Frank Staib, Peter R Galle, Andreas Teufel

**Affiliations:** 1 Department of Medicine I, Johannes Gutenberg University, Mainz, Germany

## Abstract

**Background:**

Systems biology approaches offer novel insights into the development of chronic liver diseases. Current genomic databases supporting systems biology analyses are mostly based on microarray data. Although these data often cover genome wide expression, the validity of single microarray experiments remains questionable. However, for systems biology approaches addressing the interactions of molecular networks comprehensive but also highly validated data are necessary.

**Results:**

We have therefore generated the first comprehensive database for published molecular associations in human liver diseases. It is based on PubMed published abstracts and aimed to close the gap between genome wide coverage of low validity from microarray data and individual highly validated data from PubMed. After an initial text mining process, the extracted abstracts were all manually validated to confirm content and potential genetic associations and may therefore be highly trusted. All data were stored in a publicly available database, Library of Molecular Associations http://www.medicalgenomics.org/databases/loma/news, currently holding approximately 1260 confirmed molecular associations for chronic liver diseases such as HCC, CCC, liver fibrosis, NASH/fatty liver disease, AIH, PBC, and PSC. We furthermore transformed these data into a powerful resource for molecular liver research by connecting them to multiple biomedical information resources.

**Conclusion:**

Together, this database is the first available database providing a comprehensive view and analysis options for published molecular associations on multiple liver diseases.

## Background

The completely sequenced human genome has made it possible for modern medicine to step into an era rich in genetic information and high-throughput genomic analysis [1]. Large gene expression databases [2,3] and advancing technologies in proteomics [4] provide rich sources for systemic evaluations of the development of chronic liver diseases.

These novel and readily available genetic resources and analytical tools may be the key to unravel the molecular basis of diverse chronic liver diseases as many of these must be regarded to be complex multigenic diseases. Moreover, since an efficient treatment for many of these conditions and diseases is lacking, further understanding of the genetic background of chronic liver disease will be crucial in order to develop new therapies aimed at selected targets [5-10].

At present, large genetic association studies for liver diseases are mostly based on microarray data or SAGE [11,12]. Some of these data have recently lead to the identification of prognosticly relevant subgroups in HCC suggesting that a large quantity of microarray data may aid in the identification of biologically relevant biochemical mechanisms [13-15]. However, most publicly available microarray data on chronic liver disease covers only a few samples [3]. Although these microarrays face several limitations, the data cover large expression profiles. Arguably, the biggest disadvantage is the need of confirming single microarray data by means of molecular biology, e.g. Northern Blot or RT-PCR. Single microarray experiments have been demonstrated to lack reliability with respect to validity of individual single gene expression profiles [16]. Thus, more recent microarray experiments of single probe experiments include confirmation of the proposed hypothesis by means of molecular biology. However, these experiments can be time consuming and costly. To overcome these limitations for systems biology approaches to chronic liver disease, we created a novel resource for systems biology analysis of chronic liver diseases by using PubMed published molecular associations. As multiple molecular factor genes have already been investigated in association these published studies provide a rich source of known molecular associations.

## Implementation

### Data Acquisition

In order to establish this database, the complete PubMed database, currently containing more than 17 million publications, has initially been searched by means of MeSH terms and text mining semi-automated searches [17].

Initially, for each individual disease all abstracts were searched for the disease name or respective MeSH terms providing alternative names or abbreviations which may also be used in the literature to describe the respective disease. In detail the used MeSH search strings in PubMed used for searching disease associated abstracts were:

1) "Hepatocellular" [MeSH] OR "hepatocellular carcinoma" OR "HCC" OR "hepatoma" OR "liver cancer" OR "primary liver cancer" OR "liver tumor" OR "liver carcinoma" OR "primary liver cancer" OR "hepatic tumor" for HCC

2) "biliary tract cancer" OR "gallbladder cancer" OR "cholangiocellular carcinoma" OR cholangiocarcinoma for CCC

3) "fibrosis" OR "fibroses" for liver fibrosis

4) "NASH" OR "NAFLD" OR "nonalcoholic steatohepatitis" OR "non-alcoholic steatohepatitis" OR "nonalcoholic fatty liver disease" [MeSH] for NASH

5) "AIH" OR "hepatitis, autoimmune" [MeSH] OR "autoimmune hepatitis" for AIH

6) "PBC" OR "primary biliary cirrhosis" [MeSH] OR "biliary cirrhosis, primary"

7) "PSC" OR "sclerosing cholangitis" OR "cholangitis, sclerosing" [MeSH] OR "primary sclerosing cholangitis" for PSC

The abstracts identified to be associated with the particular diseases were then searched for human, mouse, and rat gene names and alias gene names as provided by the Human Genome Organization (HUGO, http://www.hugo-international.org. Making use of the pattern matching capabilities of the Perl programming language http://www.perl.org, we used a pattern matching approach to identify gene names in the previously selected abstracts. E.g. if the gene to be searched was p53, the abstract was searched for any combinations of signs starting with the letter p followed by the numbers 5 and 3. This approach ensured a most flexible search strategy.

Mouse and rat gene names were also searched as not all authors of published abstracts went conform with the HUGO nomenclature and some of them did use murine gene names in (comparative) human studies.

By this approach we gathered a total of 101026 abstracts, potentially holding information on genetic associations to chronic liver disease. In detail we identified 44548 abstracts suggesting genetic associations for HCC, 13710 for CCC, 917 for AIH, 37173 for liver fibrosis, 2022 for NASH, 1211 for PBC and 1445 for PSC.

This strategy revealed all abstracts containing both the disease name and a gene name. However, also this semi-automated search provided a first approximation to genetic associations to liver diseases, as in multiple abstracts this genetic association could not be confirmed by reading the full abstract. E.g. the abstract may read that the gene XY is not related to disease Z, which would have also been detected by the described search strategy. Thus these automatically, by means of text mining identified abstracts, were then all individually read to confirm the suggested genetic association with the particular disease. We thereby obtained a large number of manually confirmed genetic associations to liver diseases.

Thereby, we finally identified 574 molecular associations for HCC, 150 molecular associations for liver fibrosis, 310 molecular associations for CCC, and 82 molecular associations for NASH. Only a few genes were identified to be related to the development of autoimmune liver disease: 29 abstracts describing molecular associations were found to be related to AIH, 56 to PBC, and 60 to PSC. Overall, we were able to identify a total of 1260 molecular associations for major chronic liver diseases. As all these molecular associations were manually confirmed by reading the individual full published abstract, and thus these molecular associations can be trusted to be highly reliable.

### Data organization, Webinterface

**Figure 1:**
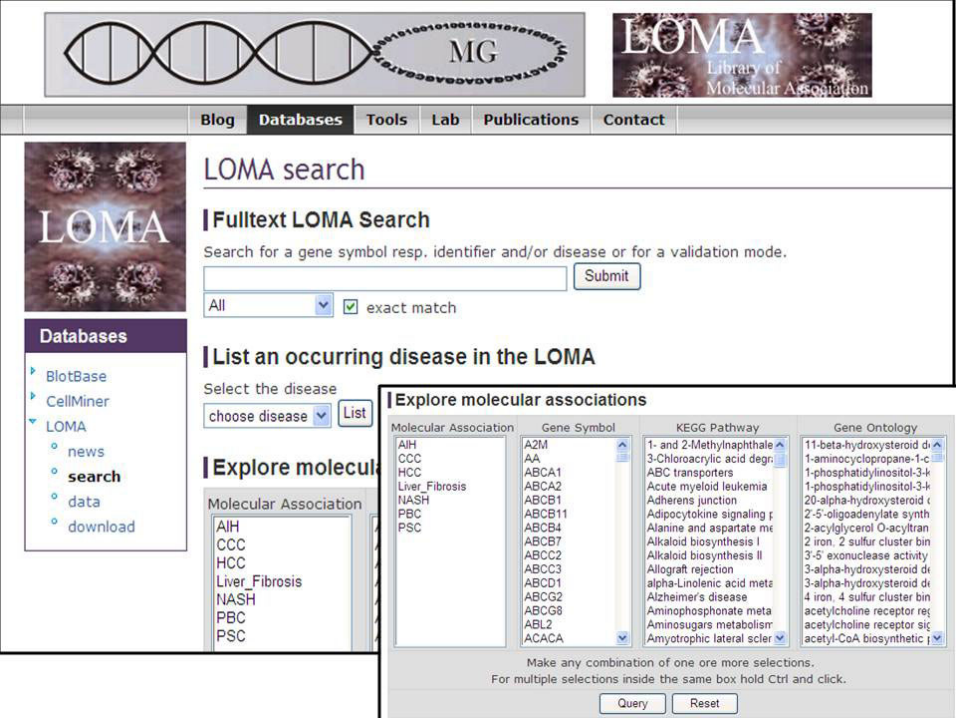
**LOMA data search interface**. LOMA offers multiple search options. Searches may be performed by means of individual gene names, NCBI Gene IDs, Ensembl Gene IDs, or disease names. Also more complex searches may be performed by selecting disease, gene symbol, a genetic pathway from KEGG, or a gene ontology from the "explore genetic association" panel.

The above described strategy of identifying potential genetic associations with chronic liver diseases identified 1260 genetic associations for several diverse chronic liver diseases. Initially the retrieved genetic associations were stored locally in a postgreSQL database http://www.postgresql.org. Subsequently, this database was then made publicly accessible and searchable through a webinterface (Figure 1) implemented in PHP http://de.php.net. It may also be downloaded as a single text file.

### Linkage to structural and functional bioinformatics information repositories

Since one of the major goals in implementing this database was to perform high throughput systems biology analyses, the LOMA genetic associations had to be linked to commonly used and established bioinformatics databases and knowledge repositories.

Gene descriptions were assembled from the NCBI Entrez database [17], chromosomal location and Ensembl ID information [18]. Furthermore, data on gene signaling and molecular pathway affiliation were collected from the Kyoto Encyclopedia of Genes and Genomes (KEGG, [19]). Finally, the Gene Ontology database was accessed to identify cellular component, biological process and molecular function information for each gene.

## Results and Discussion

### Database design and rationale

A wide variety of human diseases have been demonstrated to be genetic (inherited). Genetic mutations and a variable genetic background have been demonstrated to significantly influence the development and course of multiple diseases as well as the efficiency of treatment with diverse drugs.

Over the past decades molecular mechanisms and individual factors have been shown to be involved in the development of liver diseases and it has become clear that most liver diseases such as liver cancer, cholangiocellular carcinoma, liver fibrosis, NASH or autoimmune liver diseases are complex systemic diseases. Thus they must not only be investigated focussing on individual, potentially key regulatory genes but also with respect to underlying genetic clusters and networks [7,20-22]. However, to investigate these complex molecular interactions, data resources providing a comprehensive collection of all genes involved in the development of the diseases are urgently needed. Microarray and SAGE databases hold a vast amount of gene expression profiles [2,3]. However, the validity of individual microarray data remains low compared to data generated by means of RT-PCR, Northern-Blot, Western-Blot, RFLP, or even DNA Sequencing. As the later molecular techniques may have a higher validity they have mostly been published in individual publications, currently not available for high throughput analysis. Furthermore extracting and analyzing information on genetic associations in liver diseases already published is extremely time consuming as the respective databases may only be searched for individual publications. However, in total, these data provide a rich source of genetic information.

To overcome these obstacles, we designed a publicly available database for genetic associations with human (liver) diseases, Library of Molecular Associations (LOMA). Currently, this database holds 1260 molecular associations for a total of seven liver diseases, HCC, CCC, liver fibrosis, AIH, PBC and PSC. Most molecular associations were identified to be associated with HCC, 595, followed by CCC and liver fibrosis, 310 and 150 database entries, respectively. 82 entries were associated with the development of NASH. As expected and in concordance with a currently missing clear association of genetic networks with autoimmune liver diseases, only few genes were reported to be associated with AIH, PBC, or PSC. However, as some of these diseases, especially PBC, have been demonstrated an increased relative risk of the disease in twins and first grade relatives, a genetic basis of the disease must be suspected. Thus further research into the genetic basis of the disease is warranted to identify targets for therapeutic treatment of the disease.

In contrast to other available genetic association databases such as the Genetic Association Database [23], our database contains all published genetic associations with each specific diseases as our semi-automated search was designed to completely capture all associations.

### Database usage

The LOMA database provides multiple search options to support complex genetic analyses. Firstly, LOMA offers the option to search for individual genes and their association with different liver diseases. This search may be performed by means of a search for individual gene names, NCBI Gene IDs [17], Ensembl Gene IDs [18], or disease names. Also more complex searches may be performed by selecting disease, gene symbol, a genetic pathway from KEGG [19], or a gene ontology from the "explore genetic association" panel, providing a highly detailed search option (Figure 1).

**Figure 2:**
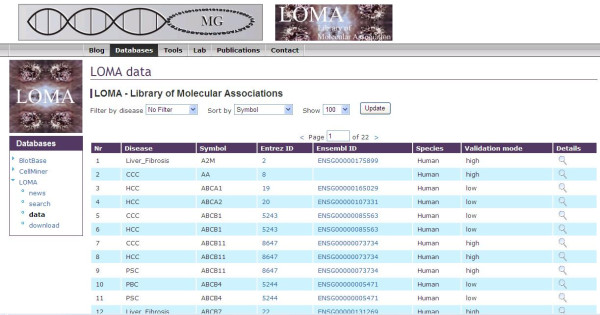
**LOMA results page**. The result page provides information on disease and individual information as well as summaries on NCBI Gene ID, Ensembl Gene ID, the species in which the molecular association to the disease was published, and number of publications reporting the molecular association ("high" stands for two or more publications). The details link provides linkage to a rich source of individual molecular information as shown in figure 3.

**Figure 3:**
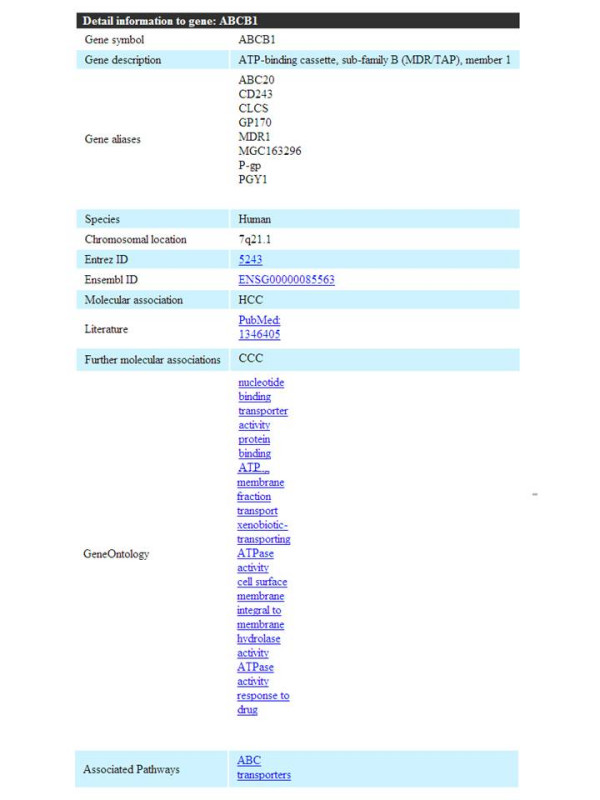
**LOMA results page**. The "Details" section of the results page provides extensive additional information and linkage to gene alias names, chromosomal location, the association documenting reference(s), gene ontology informations, and associated genetic pathways.

After executing a search, the result page for these searches offers the genetic associations to individual diseases if present. Furthermore, the results page gives a summary on gene name, associated disease, NCBI Gene ID [17], Ensembl Gene ID [18], information on the species in which the gene's association to the disease was published (if the respective gene was found to be associated with the disease in human, this category was set to "human" as default). The validation mode column gives a rough estimate, whether a genetic association was only published in a single article (low) or if the genetic association was documented in two or more articles (Figure 2). Finally, more details on the specific gene such as gene alias names, chromosomal location, the association documenting reference(s), gene ontology informations, and associated genetic pathways were provided in the "details" section (Figure 3).

For example, if one wants to know all molecular associations with the Wnt signaling pathway that have been published to play a role in HCC development, this is now easily possible with our database. On the search site under Explore molecular associations one would select "HCC" from the "Molecular Associations" column and "Wnt Signaling Pathway" from the KEGG column. The executed search will then return a number of Wnt signaling associated genes and target genes, APC, AXIN1, CTNNB1, MMP7, PRKCA, SMAD4, TP53.

For these molecular associations further information is linked especially in the details section of each gene. With this information one could for example evaluate the enrichment of the Wnt signaling pathway among all CCC related molecular factors.

#### Linkage to common bioinformatics databases

A key issue in developing this database was to provide the hepatologic community with a powerful but simultaneously highly reliable and comprehensive database to perform systems biology based high-throughput searches and comparison of gene expression, our database was linked to multiple other sources of genomic or genetic information and gene expression information in particular. This rich embedding of our database into the current scenery of bioinformatics repositories provides valuable connections which may support advanced search and evaluation strategies.

In detail, LOMA has been linked to the most commonly used bioinformatics databases, such as PubMed [17], the European Bioinformatics Institute Website Ensembl [18], the bioinformatics resource of the National Center of Biotechnology Information Entrez Gene [17], the Mouse Genome Informatics Website (MGI, [24]), and the Gene Ontology database, holding functional information on genes and proteins [25]. These links were selected as they may in addition support automated correlation with additional genomic information such as multiple sequence information, microarray expression data, conserved domains, as well as information on a gene's function.

### Comparison to other genetic association databases

Our database has been evaluated against other public databases such as Genetic Association database, HuGENavigator, or OMIM. This evaluation was performed using the molecular associations to CCC development. Comparing our text mining strategy to a manually searched sample set of 1000 randomly selected CCC associated abstracts, we documented a sensitivity of our approach of 98% and a false negative rate for abstracts not selected by or text mining approach but containing molecular associations to CCC of 2%.

For CCC development our database contained all associations also listed by other databases with two exceptions, MRP2/ABCC2 which was published only recently and the miRNA370 which was missed by our search strategy [26]. In contrast however, we provide a significantly larger list of genetic associations to CCC development of 310 molecular associations compared to 6, 19, and 39 in Genetic Association database, HuGENavigator, or OMIM, respectively.

## Conclusion

The Library of Molecular Associations (LOMA) was designed as a comprehensive database of highly reliable molecular associations conceived to close the gap between high-throughput molecular data for automated analysis and individual reliable experimental data by molecular biology. Currently this database supports information on molecular associations for several liver diseases, HCC, CCC, liver fibrosis, NASH/fatty liver disease, AIH, PBC and PSC. In addition, the database was extensively embedded into the currently available genomics repositories supporting advanced searches and cross analyses with other databases.

Together, this database is the first available database providing a comprehensive view and analysis options for published molecular associations on multiple liver diseases.

## Authors' contributions

SB, JH, TM, FS, PRG, and AT: Abstract selection and database curation. MK, AW, KS, and AT: Database programming. All authors read and approved the final manuscript.
